# Early Warning Models Using Machine Learning to Predict Sepsis-Associated Chronic Critical Illness: A Study Based on the Medical Information Mart for Intensive Care Database

**DOI:** 10.7759/cureus.67121

**Published:** 2024-08-18

**Authors:** Yulin Mei, Meng Li, Yuqi Li, Ximei Sheng, Chunyan Zhu, Xiaoqin Fan, Lei Zhang, Aijun Pan

**Affiliations:** 1 Department of Critical Care Medicine, Wannan Medical College, Wuhu, CHN; 2 Department of Intensive Care Unit, First Affiliated Hospital of Anhui Medical University, Hefei, CHN; 3 Department of Critical Care Medicine, The First Affiliated Hospital of USTC, Division of Life Science and Medicine, University of Science and Technology of China, Hefei, CHN

**Keywords:** external validation, calibration curve, predictive model, chronic critical illness, sepsis

## Abstract

Background

Patients with chronic critical illness (CCI) experience poor prognoses and incur high medical costs. However, there is currently limited clinical awareness of sepsis-associated CCI, resulting in insufficient vigilance. Therefore, it is necessary to build a machine learning model that can predict whether sepsis patients will develop CCI.

Methods

Clinical data on 19,077 sepsis patients from the Medical Information Mart for Intensive Care IV (MIMIC-IV) database were analyzed. Predictive factors were identified using the Student's *t*-test, Mann-Whitney *U* test, or *χ*^2^ test. Six machine learning classification models, namely, the logistic regression, support vector machine, decision tree, random forest, extreme gradient enhancement, and artificial neural network, were established. The optimal model was selected on the basis of its performance. Calibration curves were used to evaluate the accuracy of model classification, while the external validation dataset was used to evaluate the performance of the model.

Results

Thirty-seven characteristics, such as elevated alanine aminotransferase, rapid heart rate, and high Logistic Organ Dysfunction System scores, were identified as risk factors for developing CCI. The area under the receiver operating characteristic curve (AUROC) values for all models were above 0.73 on the internal test set. Among them, the extreme gradient enhancement model exhibited superior performance (F1 score = 0.91, AUROC = 0.91, Brier score = 0.052). It also exhibited stable prediction performance on the external validation set (AUROC = 0.72).

Conclusion

A machine learning model was established to predict whether sepsis patients will develop CCI. It can provide useful predictive information for clinical decision-making.

## Introduction

Due to the rapid technological development of the intensive care unit (ICU), the survival rate of patients in intensive care has increased. However, this has led to an increasing number of patients with chronic critical illness (CCI) relying on long-term mechanical ventilation and other critical care treatments. CCI is a devastating disease with a mortality rate comparable to that of malignancy [1,2]. Patients with CCI are prevalent in modern critical care medicine, accounting for 5-11% of the total number of ICU inpatients [1,3], while patients with CCI account for 30-50% of ICU resources [2]. The in-hospital mortality rate for patients with CCI is as high as 30.9% [3], and the five-year mortality rate is as high as 82% [4]. These statistics show that CCI imposes a huge burden on society, families, and individuals.

Pathological and physiological changes during CCI lead to persistent inflammation, immunosuppression, and catabolism syndrome (PICS), which is characterized by severe immune imbalance, nutritional metabolism disorders, and sustained damage to multiple organs [5]. Both the treatment and prevention of CCI require early intervention, including enteral nutrition, immunotherapy, bedside rehabilitation, and mechanical ventilation management [6,7]. For example, nutritional support therapy not only corrects the patient's malnutrition but also plays a role in regulating metabolic disorders, inhibiting inflammatory reactions, and regulating immune function, which have a positive impact on the outcome of CCI. At present, many clinicians have a limited understanding of CCI, and their vigilance toward CCI is relatively poor. In such cases, effective intervention in the early stages of sepsis cannot be provided to prevent CCI. This leads to high mortality rates, high medical expenses, and poor prognosis in the later stages of sepsis. Therefore, early warning of CCI is crucial. Several studies have analyzed risk factors for CCI. For instance, a prospective cohort study involving 453 patients identified mechanical ventilation, Glasgow Coma Scale <15, inadequate caloric intake, and high body mass index as independent predictors of CCI. However, owing to its small sample size and use of traditional models, its predictive performance was limited (area under the receiver operating characteristic curve (AUROC) = 0.803).

The traditional linear regression prediction method has low accuracy and poor processing ability for noisy data and is prone to overfitting when the data volume is small. In addition, linear regression methods are not suitable for nonlinear data analysis. By contrast, as a subset of artificial intelligence, machine learning can search for laws in historical data and predict future data based on those laws. Machine learning methods include clustering, classification, decision trees, Bayesian inference, neural networks, deep learning, and other algorithms [8]. With the rapid development of artificial intelligence in various fields, big data analytics can now identify the risk of disease and predict its occurrence. Previous studies on sepsis-associated CCI have focused on identifying risk factors, but few studies have used machine learning algorithms to predict CCI. Models built by machine learning algorithms can predict the occurrence of CCI, which in turn can provide guidance for early intervention. As a result, the length of stay (LOS) in the ICU may be shortened and even the mortality rate may be reduced. Therefore, machine learning models for predicting sepsis-associated CCI may have a wide range of clinical applications [4].

This study used the Medical Information Mart for Intensive Care (MIMIC)-IV database (version 2.0), which includes comprehensive clinical data from a large and diverse patient population. All datasets from different sources can be obtained in the MIMIC repository (https://mimic.physionet.org/). In this retrospective study, six machine learning models for predicting CCI, namely, logistic regression (LR), support vector machine (SVM), decision tree (DT), random forest (RF), extreme gradient enhancement (XGBoost), and artificial neural network (ANN) models, were successfully established to provide a clinical decision-making basis in potentially high-risk populations for the prevention and treatment of CCI [9].

## Materials and methods

Data sources

This study utilized data from the MIMIC-IV database (version 2.0), which contains clinical data and waveform data of bedside monitoring devices. The former includes demographic information, diagnostic information, laboratory testing information, microbial culture reports, medical imaging information, and vital sign observations, while the latter includes vital sign parameters and event records (medical measures and drugs). In addition, we utilized an independent external validation set from the MIMIC-III version 1.4 "CareVue" cohort (2001-2008). Using both databases, we carefully studied related courses and obtained permission for their use (record ID 50611006). As the MIMIC databases are approved by the institutional review boards of Beth Israel Deaconess Medical Center and MIT and all patient information in the databases is anonymized, informed consent was not required for the use of the MIMIC-IV database [10,11].

Study population and grouping

**Figure 1:**
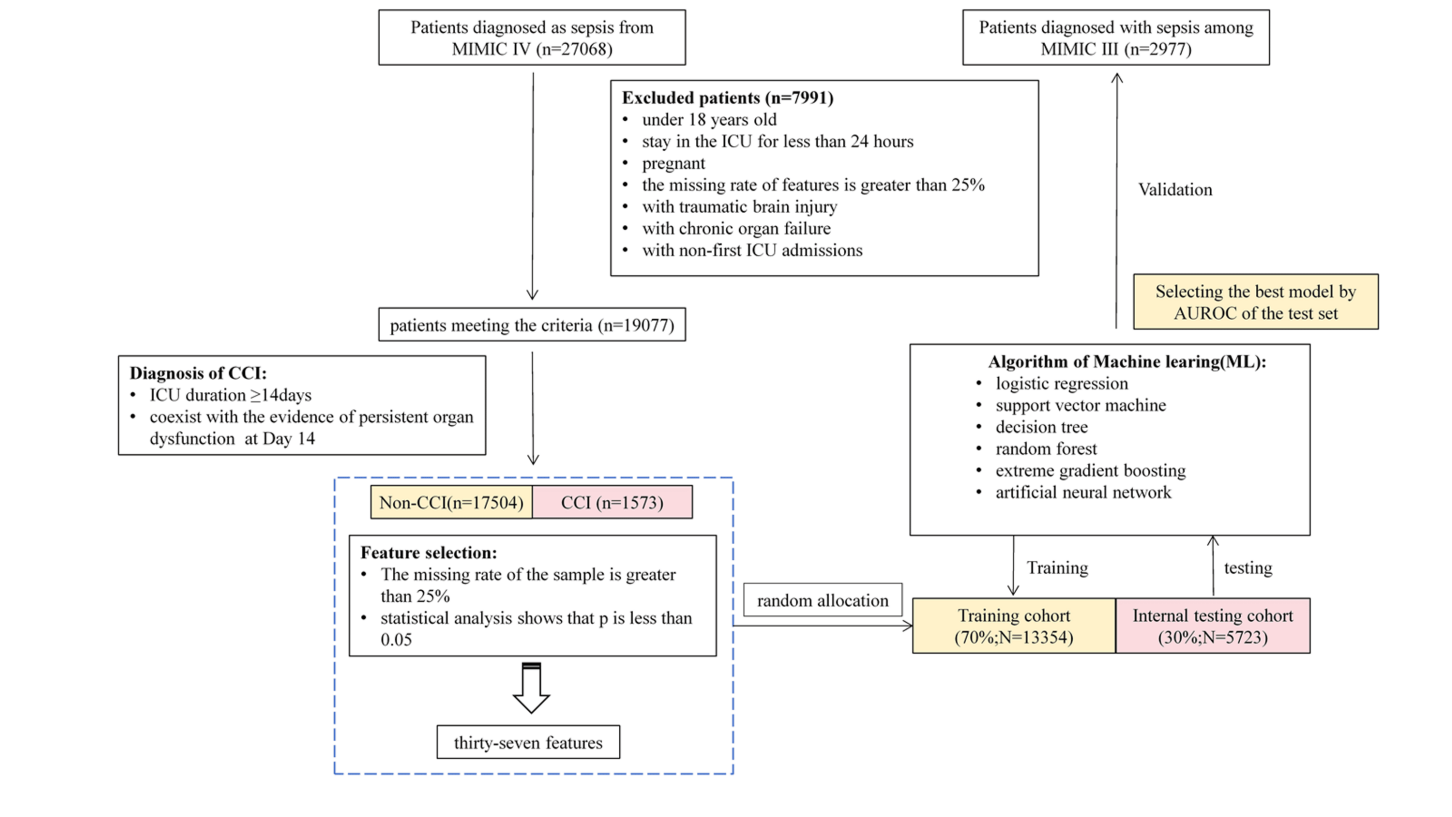
Model establishment and validation flowchart.

Inclusion criteria for this study were based on the diagnostic criteria for CCI proposed by Gardner et al. [5]: ICU hospitalization exceeding 14 days, together with persistent organ dysfunction characterized by a cardiovascular sequential organ failure assessment (SOFA) score ≥1 or a score ≥2 for any other organ. Patients were categorized into CCI and non-CCI groups based on the diagnostic criteria for CCI. We referred to diagnostic codes of the ninth revision of the International Classification of Diseases (ICD-9), available in the MIMIC-IV and MIMIC-III databases [4,12]. Exclusion criteria were as follows: patient age, <18 years; LOS in the ICU, <24 hours; pregnancy; proportion of missing data, ≥25%; craniocerebral injury (Figure 1).

Clinical variables

Several variables were extracted from the database, including demographics, vital signs, underlying disease, laboratory indicators, scoring systems, medication treatments, mechanical ventilation, extracorporeal membrane oxygenation, and renal replacement therapy. All data were collected within 24 hours of ICU admission. The outcome variable was whether patients developed CCI. In cases where the variables were measured more than once, the worst first-day laboratory variables were used.

All data collection processes were performed using PostgreSQL (version 11.19, PostgreSQL Global Development Group, USA) and Stata/MP (version 17.0, StataCorp LLC, USA).

Data filling and discretization

**Figure 2:**
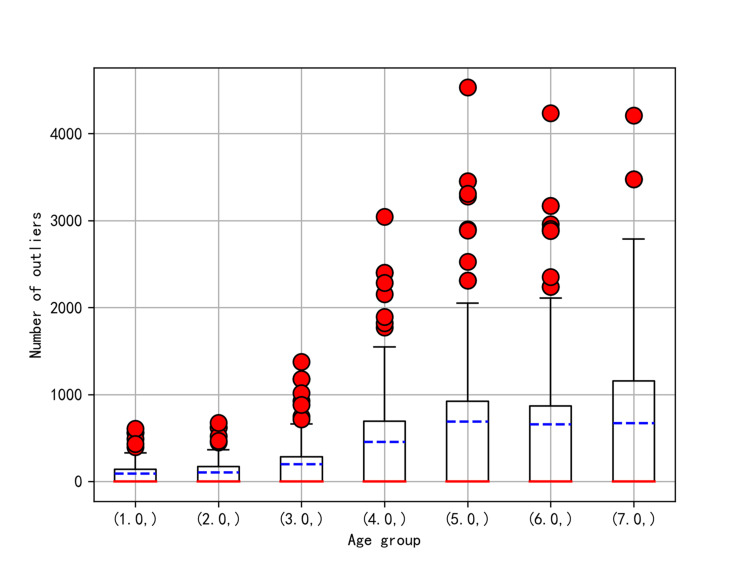
Missing-value box plot. The x-axis represents different age groups, measured in units of 10 years (1.0 represents 18 to 30 years old; 2.0 represents 30 to 40 years old, and so on). The y-axis represents the number of missing patients.

Owing to substantial variability in missing data among different age groups, segmental imputation based on age groups was not performed (Figure 2). Features with a missing rate ≥40% were excluded. Missing continuous data were imputed using the mean or median of their respective groups. There was no missing classification data and hence no need to fill it in.

**Figure 3:**
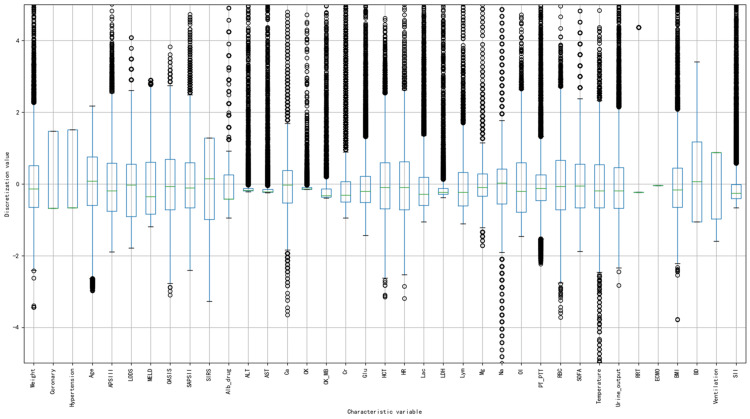
Discrete-value box plot. BMI: body mass index; BD: basis diseases; ECMO: extracorporeal membrane oxygenation; RRT: renal replacement therapy; MV: mechanical ventilation; SOFA: Sequential Organ Failure Assessment scores; SIRS: Systemic Inflammatory Response Syndrome scores; APSIII: simplified acute physiology score III; SAPSII: simplified acute physiology score II; MELD: Model for End-Stage Liver Disease scores; OASIS: Oxford acute severity of illness scores; LODS: Logistic Organ Dysfunction System scores; OI: oxygenation index; urine_output: daily urine output; ALT: glutathione aminotransferase; LDH: lactate dehydrogenase; Glu: blood glucose; CK_MB: creatine kinase isoenzyme; PTT: activated partial thromboplastin time; AST: alanine aminotransferase; CK: creatine kinase; RBC: red blood cell; HCT: hematocytometry; SII: systemic inflammatory response index; Alb_drug: albumin dosage within 24 hours

Data discretization enhances data mining efficiency and accuracy, optimizes algorithms for filtering noise and identifying outliers, and mitigates overfitting. Therefore, we converted 41 continuous features (e.g., glucose, Na+ concentration, heart rate, diastolic blood pressure, and urea) into discrete data. For instance, Na+ concentrations were classified into hypoosmolar, iso-osmolar, and hyperosmolar based on thresholds of 135 mEq/L and 145 mEq/L. Heart rate was categorized as bradycardia, normal, and tachycardia using thresholds of 60 beats/min and 100 beats/min. This method focused particularly on features with a high prevalence of outliers. We constructed a box plot encompassing all features and manually established the range for normal values (mean ± 2.5 * standard deviation). Values beyond this range were designated as outliers, each denoted by an open circle (Figure 3).

Construction and evaluation of predictive models

Feature Selection

Student's t-test, the Mann-Whitney U test, and the χ2 test were used to screen for statistically significant factors between groups.

Data Partitioning

The patients were randomly divided into training and testing sets in a 7:3 ratio. The training set comprised 13,353 individuals, and the testing set comprised 3,473 individuals. Considering the markedly smaller number of positive samples compared with the number of negative samples, positive samples in the training set were oversampled to match the number of negative samples before building the model.

Comprehensive Analysis of Classification Models

Using the sklearn library (version 1.3.2) in Python, multiple classification models were developed, including LR, SVM, DT, RF, XGBoost, and ANN. The training set underwent fivefold cross-validation, followed by evaluation using the testing set to assess the predictive performance of various models. Calibration curves were generated using Python (sklearn, version 1.3.2) to further assess the models' predictive accuracy. Subsequently, a comprehensive evaluation of predictive models was conducted to validate their usefulness in decision support. The optimal model was ultimately selected.

External Validation

Further evaluation of model performance was conducted using an external validation set from MIMIC-III.

Statistical analysis

Continuous variables that followed a normal distribution are represented as mean ± standard deviation and were compared using Student's t-test. Non-normally distributed data are represented as median (interquartile range) and were compared using the Mann-Whitney U test. To balance the large number of negative samples, random sampling was used to select an equivalent number of negative samples for statistical analysis alongside the positive samples. Each feature underwent 1,000 iterations of testing, and the resulting average value was reported as the p-value. A threshold of p < 0.05 was considered statistically significant. Statistical analyses were performed using IBM SPSS Statistics for Windows, version 27.0.1 (released 2020, IBM Corp., Armonk, NY) (and Python (version 3.10.9, Python Software Foundation, USA).

For a detailed introduction to the used code, see https://github.com/15990036592/1.git.

## Results

Baseline data comparison

Table 1 presents the fundamental characteristics of the study population. After excluding patients with substantial missing data, the study included 19,077 sepsis patients from the MIMIC-IV dataset, of which 1,573 (8.2%) were classified as CCI patients. After statistical testing, 37 statistically significant characteristic variables were selected, including age, aspartate aminotransferase (AST), and systemic immune inflammation index.

**Table 1:** Baseline characteristics in the positive cohort and negative cohort. CCI: chronic critical illness; IQR: interquartile range; SD: standard deviation; BMI: body mass index; BD: basis diseases; ECMO: extracorporeal membrane oxygenation; RRT: renal replacement therapy; MV: mechanical ventilation; SOFA: Sequential Organ Failure Assessment scores; SIRS: Systemic Inflammatory Response Syndrome scores; APSIII: Simplified Acute Physiology Score III; SAPSII: Simplified Acute Physiology Score II; MELD: Model for End-Stage Liver Disease scores; OASIS: Oxford acute severity of illness scores; LODS: Logistic Organ Dysfunction System scores; OI: oxygenation index; urine_output: daily urine output; ALT: glutathione aminotransferase; LDH: lactate dehydrogenase; Glu: blood glucose; CK_MB: creatine kinase isoenzyme; PTT: activated partial thromboplastin time; AST: alanine aminotransferase; CK: creatine kinase; RBC: red blood cell; HCT: hematocytometry; SII: systemic inflammatory response index; Alb_drug: albumin dosage within 24 hours

Characteristic	CCI (n = 1,573)	非CCI (n = 17,504)	P-value
Age (years), mean (SD)	63 (15)	67 (15)	<0.001
Gender (male)(%)	895 (56.9%)	10,389 (59.4%)	0.061
Weight (kg), median (IQR)	83 (68.99)	80 (68.94)	<0.001
BMI (kg/m^2), median (IQR)	29.3 (24.8, 32.9)	29.3 (25.8, 29.8)	0.005
Hypertension (%)	373 (23.7%)	5412 (30.9%)	<0.001
Coronary (%)	323 (20.5%)	5729 (32.7%)	<0.001
BD (times), mean median (IQR)	1 (0,1)	1 (0, 2)	<0.001
ECMO (%)	14 (0.9%)	24 (0.13%)	<0.001
RRT (%)	140 (8.9%)	810 (4.6%)	<0.001
MV (%)	1,315 (77.5%)	9948 (48.7%)	<0.001
SOFA, mean (SD)	7 (4)	6 (3)	<0.001
SIRS, median (IQR)	3 (3,4)	3 (2, 3)	<0.001
APSIII, mean (SD)	80 (25)	52 (22)	<0.001
SAPSII, mean (SD)	44 (14)	39 (13)	<0.001
MELD, median (IQR)	15 (9,22)	13 (9, 20)	0.005
OASIS, mean (SD)	43 (8)	35 (9)	<0.001
LODS, mean (SD)	9 (3)	6 (3)	<0.001
Heart rate (times/min), mean (SD)	110 (21)	105 (19)	<0.001
Temperature (celsius), median (IQR)	37.6 (37.2, 38.2)	37.4 (37.1, 37.9)	<0.001
PH, median (IQR)	7.41 (7.38, 7.46)	7.42 (7.39, 7.45)	0.548
OI (mmHg), median (IQR)	128 (84, 184)	168 (127, 200)	0.001
Urine_output (ml), median (IQR)	1387 (810, 2115)	1625 (1005, 2335)	<0.001
Magnesium (mg/dL), median (IQR)	2.2 (2.0, 2.5)	2.2 (2.0, 2.4)	0.034
Creatinine (mg/dL), median (IQR)	1.2 (0.8, 1.8)	1.1 (0.8, 1.6)	0.002
ALT (U/L), median (IQR)	37 (27, 53)	34 (28, 40)	0.003
LDH (U/L), median (IQR)	458 (382)	348 (255)	<0.001
Glu (mg/dL), median (IQR)	168 (134, 214)	158 (128, 195)	<0.001
Sodium (mEq/L), median (IQR)	141 (138, 143)	140 (137, 142)	0.015
Ck_MB (U/L), median (IQR)	6.2 (16.2)	3.9 (11.1)	0.002
Lactate, median (IQR)	2.1 (1.2, 3.8)	1.8 (0.8, 2.8)	<0.001
PTT (s), median (IQR)	35.8 (28.9, 47.9)	33.8 (28.9, 43.4)	0.010
Calcium (mg/dL), median (IQR)	8.4 (7.9, 8.8)	8.4 (8.0, 8.7)	0.008
AST (U/L), median (IQR)	61 (44, 94)	51 (43, 63)	<0.001
CK (U/L), median (IQR)	730 (1618)	464 (1374)	0.001
RBC (10^12/L), mean (SD)	3.3 (0.7)	3.3 (0.7)	0.003
HCT (%), median (IQR)	33.8 (29.9, 38.0)	33.0 (29.5, 36.7)	<0.001
Lymphocytes (%), median (IQR)	10.6 (4.5)	12.0 (4.9)	0.016
SII (10^9), median (IQR)	1502 (984, 2382)	1284 (822, 2067)	0.014
Alb_drug (g), median (IQR)	7.9 (16.9)	5.9 (13.0)	0.027

Comprehensive model analysis

**Figure 4:**
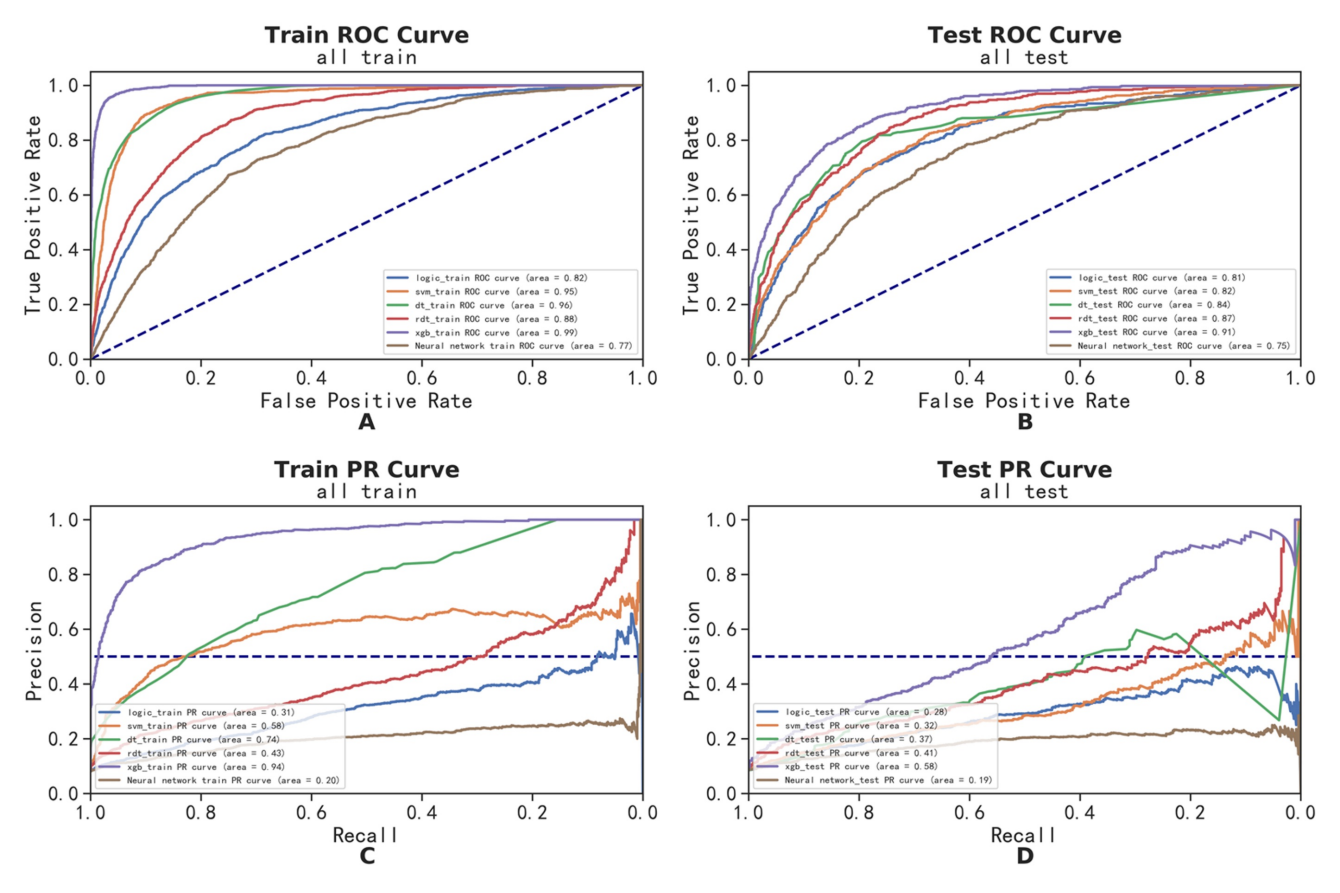
Machine learning models' comprehensive analysis. (A) Train receiver operating characteristic (ROC) curve. (B) Train precision-recall (PR) curve. (C) Test ROC curve. (D) Test PR curve. The ratio of the training set to the test set is 7:3.

We developed six machine learning models, namely, LR, SVM, DT, RF, XGBoost, and ANN. The area under the receiver operating characteristic curve (AUROC) for each of the six models was used to compare model performance on both training and testing sets. A larger AUROC indicates stronger discriminative ability (Figure 4A, 4C) [13,14]. Classification reports are summarized in Table 2. In the test set, except for the ANN, the AUROCs of all models were above 0.8, and the XGBoost model performed the best (AUROC = 0.91, accuracy = 0.93, specificity = 0.93, sensitivity = 0.93). While the AUROC evaluates prediction accuracy, precision-recall curves are more clinically applicable [15]. In the training and testing sets, XGBoost still showed the best performance, having the highest average precision (AP) values of 0.90 and 0.58, respectively (Figure 4B, 4D). Altogether, XGBoost was proven to be the optimal model.

**Table 2:** Predictive performance of models in the training and test sets. LR: logistic regression, SVM: support vector machine, DT: decision tree, RF: random forest, XGBoost: extreme gradient boosting, ANN: artificial neural network, AUROC: area under the receiver operating characteristic curve

Model	Training set					Test set				
	Accuracy	Specificity	Sensitivity	F1-score	AUC	Accuracy	Specificity	Sensitivity	F1-score	AUC
LR	0.90	0.95	0.88	0.89	0.82	0.89	0.95	0.87	0.88	0.81
SVC	0.95	0.99	0.92	0.93	0.95	0.89	0.95	0.86	0.87	0.82
DT	0.94	0.96	0.95	0.94	0.96	0.91	0.94	0.92	0.91	0.83
RF	0.91	0.93	0.92	0.90	0.88	0.90	0.93	0.92	0.90	0.86
XGBoost	0.94	0.94	0.94	0.92	0.99	0.93	0.93	0.93	0.91	0.91
ANN	0.89	0.94	0.88	0.89	0.73	0.90	0.93	0.92	0.90	0.72

**Figure 5:**
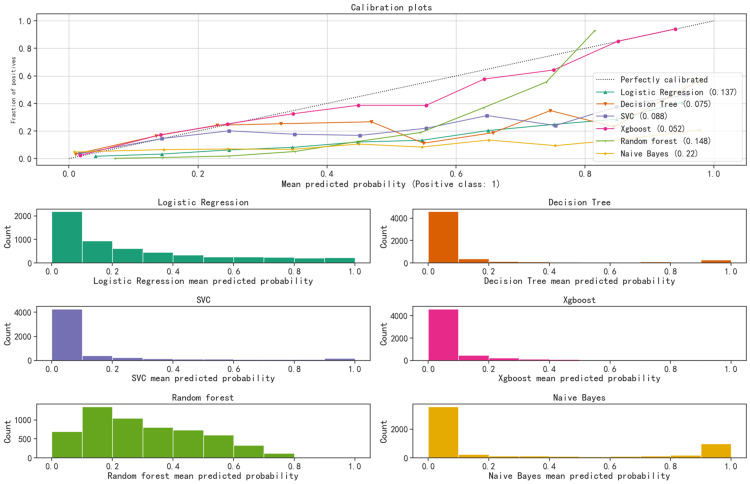
Calibration curve of the test set. The x-axis represents the average predicted probability, and the y-axis represents the actual probability of the event. The dashed diagonal line serves as the reference line. The other smooth solid lines represent different model fitting lines. The closer a fitting line is to the reference line, the more accurate the model's prediction, as indicated by the smaller values in brackets.

The calibration curve for the testing set plots the mean predicted probability on the horizontal axis and the actual probability of events on the vertical axis. A dashed diagonal line serves as a reference, while smooth solid lines represent the fits of different models. A closer fit to the reference line and a smaller value in parentheses indicate that the model's predicted values were more accurate. The calibration curve highlights that both XGBoost and RF models achieved higher accuracy. The Brier score measures the mean squared error between predicted probabilities and actual outcomes. The Brier scores for the XGBoost and RF models were 0.052 and 0.148, respectively (Figure 5).

**Figure 6:**
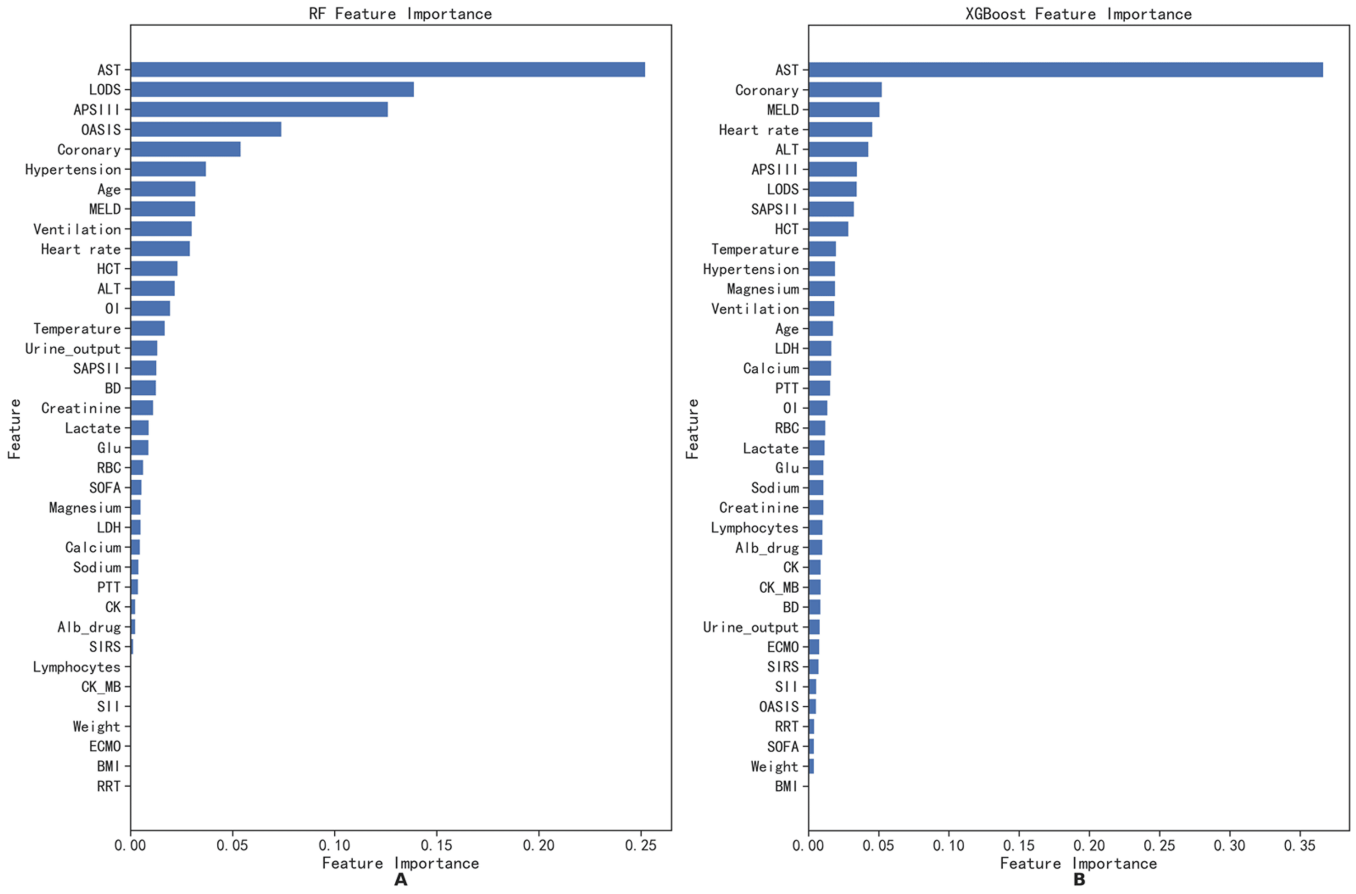
Feature importance ranking of extreme gradient enhancement (A) and random forest (B). The horizontal axis represents the weight size of each feature, and the vertical axis represents the ranking of feature importance. BMI: body mass index; BD: basis diseases; ECMO: extracorporeal membrane oxygenation; RRT: renal replacement therapy; MV: mechanical ventilation; SOFA: Sequential Organ Failure Assessment scores; SIRS: Systemic Inflammatory Response Syndrome scores; APSIII: Simplified Acute Physiology Score III; SAPSII: Simplified Acute Physiology Score II; MELD: Model for End-Stage Liver Disease scores; OASIS: Oxford acute severity of illness scores; LODS: Logistic Organ Dysfunction System scores; OI: oxygenation index; urine_output: daily urine output; ALT: glutathione aminotransferase; LDH: lactate dehydrogenase; Glu: blood glucose; CK_MB: creatine kinase isoenzyme; PTT: activated partial thromboplastin time; AST: alanine aminotransferase; CK: creatine kinase; RBC: red blood cell; HCT: hematocytometry; SII: systemic inflammatory response index; Alb_drug: albumin dosage within 24 hours

**Figure 7:**
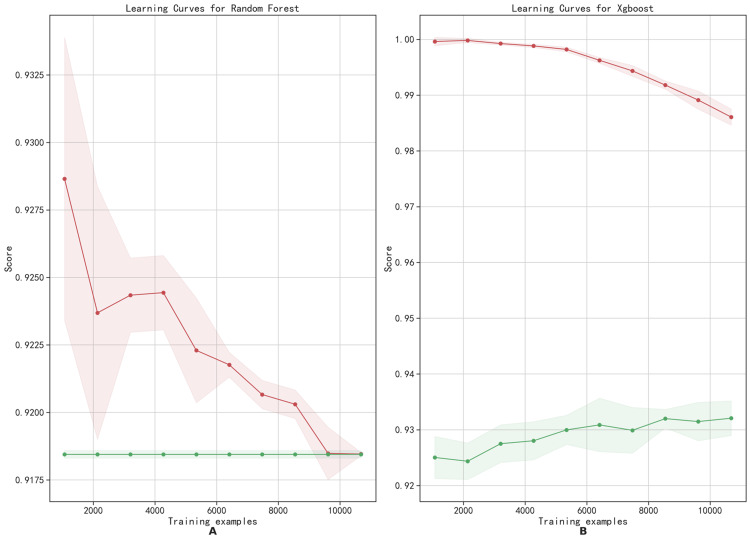
Training history curve of extreme gradient enhancement (A) and random forest (B). The horizontal axis represents the number of training samples, and the vertical axis represents the accuracy score. The red curve represents the training set, and the green curve represents the test set.

On the testing set, the RF clearly outperformed other models, achieving AUROC and AP values of 0.88 and 0.40, respectively. We conducted a comparative analysis between RF and XGBoost, plotting feature importance rankings (Figure 6A, 6B), training history curves (Figure 7A, 7B), and tree diagrams. Key features in the RF model included AST, Acute Physiology and Chronic Health Evaluation III (APACHE III), and Logistic Organ Dysfunction System, while those in the XGBoost were AST, Model for End-Stage Liver Disease, and coronary heart disease. In the training history curves, the x-axis quantifies the number of training samples, and the y-axis quantifies the accuracy scores (Figure 6). As the sample size increased, accuracy decreased for both models on training sets, contrasting with minor fluctuations in validation set accuracy. A small training set yields low training error but high cross-validation error, indicating potential overfitting. Larger training sets enhance model generalization, stabilizing errors in both sets. These curves elucidate the relationship between the model performance and training set size, which facilitates model selection, optimization, and determination of whether more data are needed to improve performance. The XGBoost model exhibited a more pronounced disparity between training and validation accuracies than the RF model, suggesting a proclivity for overfitting. Conversely, the RF model demonstrated a superior balance between training and validation accuracies, indicating its robust generalization capability.

External validation and model evaluation

**Figure 8:**
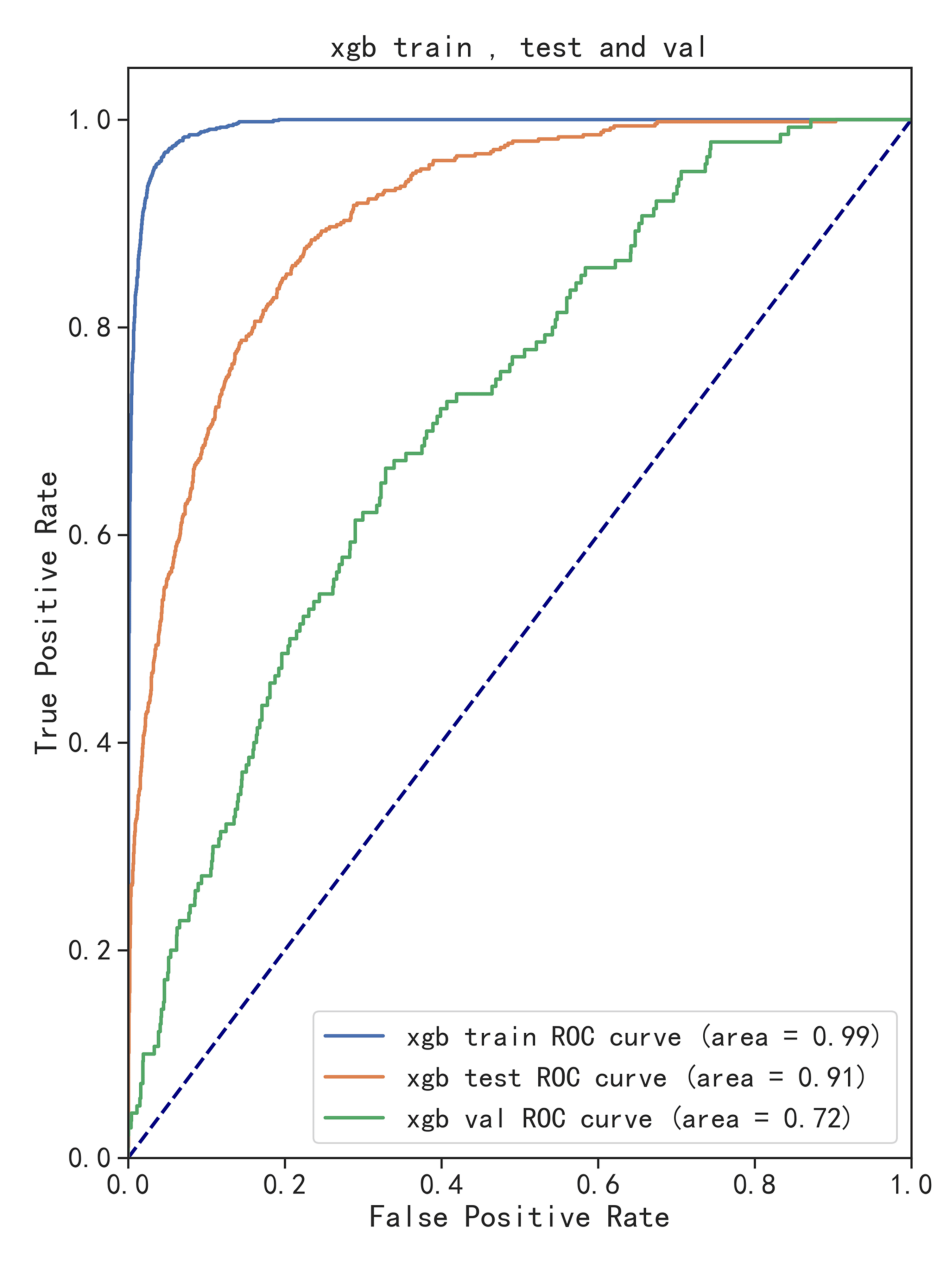
Model performance of extreme gradient enhancement on training, testing, and validation sets. Blue represents the training set; yellow represents the testing set, and green represents the validation set.

To further validate the generalizability of our model and mitigate its inherent potential for overfitting, we used data on 2,977 sepsis patients obtained from MIMIC-III version 1.4. Our best-trained model was validated using this independent dataset, demonstrating that the XGBoost model maintained strong predictive performance (AUROC = 0.72) using external data (Figure 8).

## Discussion

In our study, six machine learning models were used. By analyzing the ROC and PR curves, we found that the XGBoost model performed better than other models on the test set (AUROC = 0.91 and AP = 0.58). Generally, an AUROC of 0.7 is considered a reasonable threshold indicating good predictive performance of a model, and an AUROC > 0.8 indicates excellent model performance [16]. However, it should be noted that the AUROC cannot solely explain the performance of the model. It is also necessary to combine other indicators, such as specificity, sensitivity, and F1 score, for comprehensive evaluation. Except for LR and SVM models, all other models have specificity > 0.93, sensitivity > 0.92, and F1 score > 0.90. The XGBoost model also performed excellently in these three aspects (specificity = 0.93, sensitivity = 0.93, F1 score = 0.91), indicating that this model can reduce misdiagnosis and missed diagnosis, thereby improving the credibility of the medical system [17].

Approximately 8.2% of sepsis patients registered in the internal dataset from MIMIC-IV progressed to CCI, indicating that CCI is a rare event. The XGBoost model uses custom loss functions, regularization, and weighted voting mechanisms to handle imbalanced data. The RF model processes imbalanced data and evaluates feature importance through ensemble learning, providing stable and accurate predictions for low-probability events [18,19]. Therefore, the XGBoost and RF models excel in predicting rare events, and our results also confirm this point.

There are 27,068 sepsis patients registered in the MIMIC-IV database. However, some patients are not suitable for inclusion in our study. First, pregnant women were excluded because they may differ significantly from general sepsis patients in terms of drug resistance, contraindications, and treatment plans. Second, because traumatic brain injury can also lead to CCI, patients with such injury were also excluded. Third, considering the negative impact of excessive missing data on model efficiency, such patients were excluded. Lastly, considering the inherent impact of chronic organ failure on SOFA scores, patients with chronic organ failure were excluded. Before model establishment, 7,991 patients were excluded, which may have helped us to avoid the bias caused by the above factors, improve model performance, reduce overfitting, and ultimately create a more reliable model.

Feature selection is crucial in machine learning. Selecting the most relevant feature from a large number of potential features is of great significance for enhancing interpretability and avoiding overfitting [20]. From 66 features, we selected 37 clinical feature variables with significant differences using statistical tests. Therefore, lasso regression was not used to eliminate more features, but the importance score for each feature was evaluated using each algorithm, which will weigh all features before building the model. This is first because lasso regression performs better on high-dimensional data, which contain many more features than the sample size, but its advantage is not marked for low-dimensional data, which contain relatively few features. Second, lasso regression may erroneously remove some useful features, leading to bias in the results. Finally, embedded methods such as DT, RF, and XGBoost automatically perform feature selection during model training. These methods not only consider the importance of features but also simultaneously perform feature selection and model training [20,21]. In our study, the performance of the model established using lasso regression to screen variables was worse than that without lasso regression (data not shown).

AST emerged as the top factor in feature importance rankings using the XGBoost and RF models. AST is an enzyme widely present in the liver, heart, muscles, and other tissues, and elevated AST levels typically indicate liver cell damage or inflammation. The liver releases many inflammatory cytokines during injury, such as tumor necrosis factor-alpha and interleukin-1 beta, which can cause overexpression of surface molecules in antigen-presenting cells, thereby inducing a wide range of inflammatory responses through systemic circulation [22]. AST also reflects metabolic stress linked to insulin resistance and lipid metabolism disorders. Its main function is to catalyze the amino transfer reaction between aspartic acid and alpha-ketoglutarate, producing oxaloacetic acid and glutamic acid. Its excessive release into the bloodstream can cause abnormalities in glucose metabolism, lipid metabolism, and protein metabolism and even lead to internal metabolic disorders in the myocardium and skeletal muscles [23]. Therefore, high AST is not only a marker of liver injury but also plays an important role in PICS.

External validation is crucial for assessing model performance and generalizability. It ensures models handle unseen data in real-world applications and also develops reliable machine learning systems effectively. We used MIMIC-III as the external validation set because the time ranges covered by the MIMIC-IV and MIMIC-III databases are different, which helps to evaluate a model's generalization ability across different time periods, data structures, and patient populations. Therefore, the MIMIC-III database is very suitable for external validation of models developed using the MIMIC-IV database. The results showed that the XGBoost model also demonstrated good performance using external validation sets (AUROC = 0.72), highlighting its effectiveness in generalization beyond training data.

Several limitations of our study should be mentioned. Data from a single public database limits the generalizability of the results. Variables, such as CRP, albumin, and lymphocytes, which may be closely related to the development of CCI, were excluded because these features had too many missing values [24]. Finally, all data were collected within 24 hours after ICU admission, and we did not record data by hour or day for real-time analysis of patient physiological data. Future research may combine specific features to establish time-series models, aiming to establish dynamic warning models for predicting CCI.

## Conclusions

In summary, we developed a machine learning model that demonstrated optimal performance on the internal test set and maintained robust performance on the external validation set. The model exhibits strong interpretability and predictive capability, making it suitable for predicting whether sepsis patients will develop CCI. This provides valuable predictive information for clinical decision-making.
